# Nutrient export from Finnish rivers into the Baltic Sea has not decreased despite water protection measures

**DOI:** 10.1007/s13280-019-01217-7

**Published:** 2019-07-05

**Authors:** Antti Räike, Antti Taskinen, Seppo Knuuttila

**Affiliations:** Finnish Environment Institute, Latokartanonkaari 11, 00790 Helsinki, Finland

**Keywords:** Baltic Sea, Nitrogen, Phosphorus, Point sources, Riverine export, Water protection targets

## Abstract

To tackle the symptoms of eutrophication in the open Baltic Sea and Finnish coastal waters, Finland has agreed to reduce both total nitrogen (TN) and total phosphorus (TP) inputs. Due to large investments in treatment of municipal and industrial wastewaters, TP loads started to decrease already in the mid-1970s and the respective TN loads in the mid-1990s. During the last two decades, much effort has been spent in decreasing the load originating from diffuse sources. Trend analyses in 1995–2016 showed that, despite various mitigation measures, riverine nutrient export has not substantially decreased, and especially the export from rivers draining agricultural lands remains high. In some areas TN concentrations and export were increasing and we found evidence that it was linked to ditching of peatlands. Several factors connected to climate/weather (e.g. temperature and precipitation) have counteracted the mitigation measures, and therefore Finland will not achieve the nutrient reduction targets by 2021.

**Electronic supplementary material:**

The online version of this article (10.1007/s13280-019-01217-7) contains supplementary material, which is available to authorized users.

## Introduction

Despite the measures taken to reduce external inputs of nitrogen (N) and phosphorus (P) to the Baltic Sea, good ecological status has not been reached and nearly the entire sea area is still affected by eutrophication (HELCOM 2018). The deterioration of water quality is also commonly detected in Finland’s coastal and marine waters, and the state of the coastal waters in southern Finland is particularly poor (HELCOM 2018).

In order to tackle the symptoms of eutrophication, the member countries of the Helsinki Commission (HELCOM) have agreed to decrease the nutrient inputs to the Baltic Sea. The nutrient reduction scheme of the HELCOM Baltic Sea Action Plan (BSAP) was revised in 2013, and now the needed reductions are 118 000 t total nitrogen (TN) and 15 200 t total phosphorus (TP) on an annual basis by the 2021 deadline (http://www.helcom.fi/baltic-sea-action-plan/nutrient-reduction-scheme/targets). These correspond to approximately 13% of the mean TN inputs and 41% of the mean TP inputs in the reference period 1997–2003. The respective reduction targets for Finland are 3030 t (4%) of the TN inputs and 356 t (10%) of the TP inputs. Besides BSAP, several European Union (EU) directives aim at improving the state of marine environments. Two central directives in this respect are the Water Framework Directive (WFD), which aims at achieving good ecological and chemical status for inland and coastal surface waters (WFD 2000), and the Marine Strategy Framework Directive (MSFD) aiming at achieving or maintaining a good environmental status of European marine waters by 2020 (MSFD 2008). In order to reach a good ecological and chemical status, countries should implement the WFD’s regional River Basin Management Plans (RBMPs), which define the measures needed to achieve this target. According to the RBMPs, Finland should reduce annual TN loads into the coastal waters by 6600 t and the respective TP loads by 440 t of the mean inputs in the reference period 2006–2011 (Laamanen 2016).

Eutrophication-related water quality policy in Europe and the USA has been directed primarily towards P control for freshwater ecosystems (Wong et al. 2018). Nutrient reduction measures in Finland have also been targeted at P removal since Finnish inland waters have been generally regarded as P limited (Räike et al. 2003). In Finland, P removal from municipal wastewater started in the mid-1970s, though in the pulp and paper industry, Finland’s biggest industrial sector, such measures were not taken until the late 1980s. In 2014 the P removal for the whole country was 93% (including municipal and industrial wastewater). Due to efficient P removal from municipal and industrial wastewater, the relative importance of diffuse inputs has increased during the last few decades. Presently, point sources comprise less than 15% of the Finnish nutrient inputs to the Baltic Sea.

**Fig. 1:**
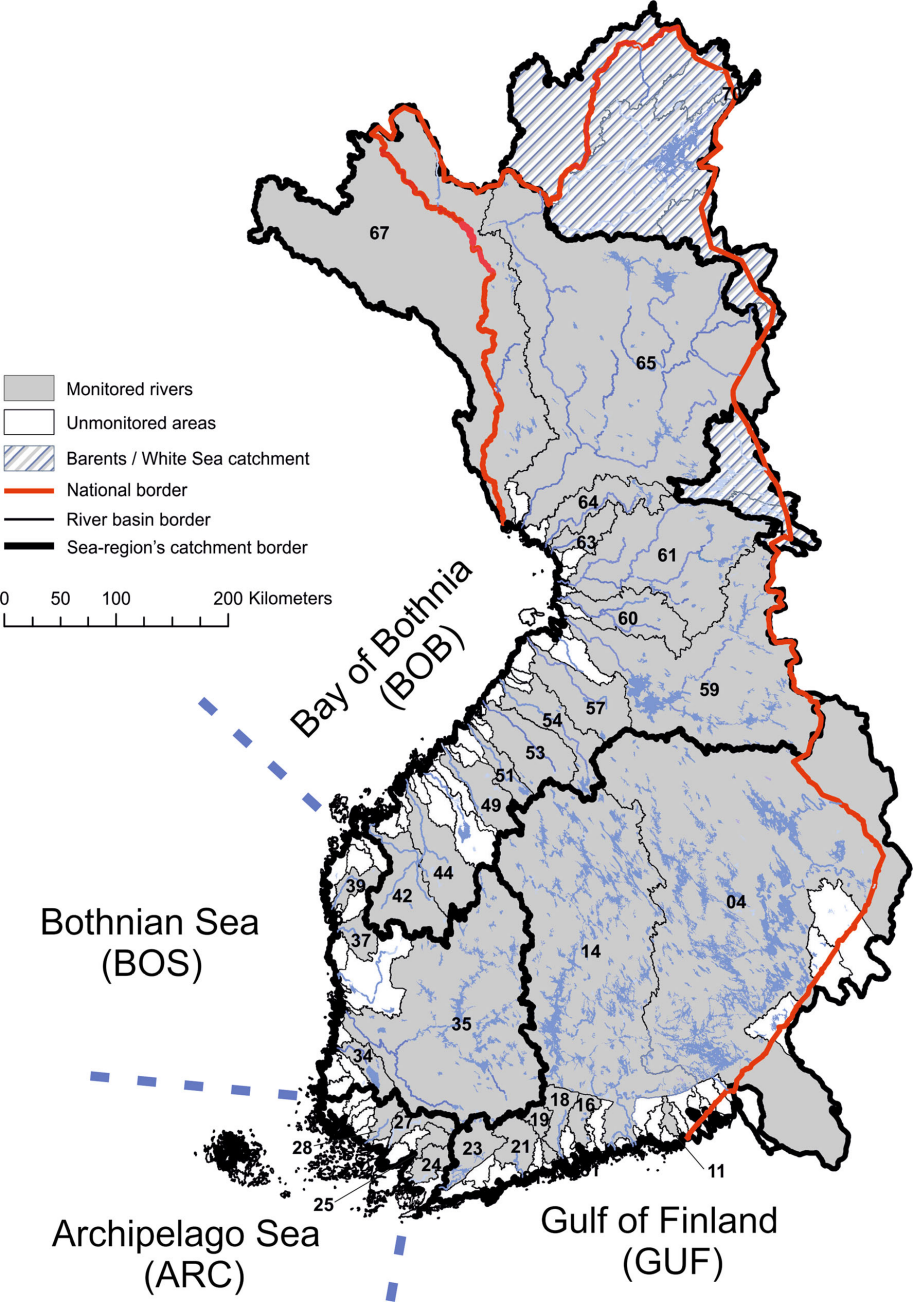
Map of Finland showing monitored rivers and unmonitored areas of the Baltic Sea catchment. The shaded areas are part of the Barents Sea or White Sea catchment

N is estimated to be the limiting nutrient in the Finnish sea regions south of the Bothnian Bay (BOB; Tamminen and Andersen 2007). N removal from municipal wastewaters was started in the mid-1990s in some larger municipal treatment plants discharging directly to the Gulf of Finland (GUF). In 2014 the N removal for the whole country was 38% (including municipal and industrial wastewater). According to Finland’s national implementation plan of the EU MSFD, the N removal efficiency should be increased to at least 70% in all those municipal treatment plants south of the BOB that have a population equivalent of > 10 000 and discharge directly into coastal waters (Fig. 1). Furthermore, it should be improved to 90% for larger treatment plants which discharge into coastal waters, whenever this is technically and economically feasible. Most of the achievable N reduction in the point source load can be gained through improvements in the municipal waste water treatment plants (WWTPs).

Anthropogenic nutrient pools can be divided, beside direct discharges into coastal waters and rapidly transported pool (e.g. surface runoff and erosion), into stable legacy pools (strongly bound forms in soil) and mobile legacy pools (loosely bound forms in soils; McCrackin et al. 2018). Rivers’ transport constitutes the major part of the N and P inputs to the Baltic Sea (HELCOM 2011), and beside the Baltic Sea, riverine nutrient export has increased globally (Seitzinger et al. 2010). This increase has taken place also outside the Northern Hemisphere, e.g. Africa (Yasin et al. 2010), Asia (Pedde et al. 2017) and South America (Van Der Struijk and Kroeze 2010). Recent Finnish studies indicate that TP concentrations and flow-normalised TP export into the Baltic Sea from Finnish rivers have decreased, but TN concentrations and flow-normalised TN export have increased (Ekholm et al. 2015; Rankinen et al. 2016). The rivers included in these studies flow through cultivated catchments, which are important contributors of TN and TP loads into the sea, but their catchments cover only 15% of the total Finnish Baltic Sea catchment area. In this study, we examined trends in TN and TP concentrations and flow-normalised and non-normalised TN and TP export into the Baltic Sea by all Finnish rivers in 1995–2016. Since the BSAP nutrient reduction targets are divided between the Baltic Sea’s sub-regions, we also studied trends by sub-regions. Furthermore, we studied whether the water protection measures taken so far have been effective in reducing nutrient loading from Finland into the Baltic Sea and assessed whether the Finnish nutrient reduction targets will be fulfilled by the 2021 deadline. The focus was on the period from 1995 onwards, as that was the year when Finland joined the EU and the first Agri-Environment Programme was launched.

## Materials and methods

### Catchment properties

The total catchment area of the studied rivers was 332 000 km^2^ (296 000 km^2^ was monitored), which apart from Finnish territory also includes the transboundary catchment areas of Sweden, Norway and Russia that carry water into Finnish territory (Fig. 1). The average proportion of forests (based on CORINE Land Cover 2012 25 × 25 m grids) was 47% (range 33–54%) and that of peatlands 18% (3–40%) (Table S1). The percentage of peatlands is highest at latitudes between 63° and 66°N, whereas the share of forests increases towards the south. The proportion of agricultural land in the river basins was on average 7% (1–43%). The majority of cultivated areas are located close to the southern and western coasts. The average proportion of water coverage of the catchments was 10% (0.5–19%) and the average coverage of urban areas was 3% (1–20%).

The mean annual flow of the rivers varied from 4 to 622 m^3^ s^−1^, and the annual runoff from 245 to 427 mm (Table S1). The runoff was usually higher in the northern parts of the country where evaporation is lower. Spring peak-flow normally occurs in April in the southern and central parts of the country and in May in the northern regions. More detailed information of the location of sampling stations and basic catchment characteristics can be found in Räike et al. (2012).

### Data sources and analytical methods

Point source loads, water quality and water flow data from 1995 to 2016 were obtained from the national databases maintained by the Finnish Environment Institute (SYKE). The sampling depth varied between 0 and 2 m. The total number of analyses was 25 100 (Table S2). The median annual sampling frequency was 12 (5–20 in individual rivers in 2016). Sampling was conducted at monthly intervals, except in rivers draining agricultural areas in southern Finland in which nutrient concentrations vary more widely depending on changes in flow. In those rivers sampling frequency was 22–58 (Table S2) and extra samples were taken especially on high flow events. In addition to TN and TP, we also studied changes in their soluble fractions (nitrate, NO_2,3_-N; ammonium, NH_4_-N; phosphate, PO_4_-P) and total suspended solids (TSS) as supplementary variables. Nutrients were analysed from unfiltered samples by Finnish standard methods. The only exception was PO_4_-P, which was filtered like TSS, with Nucleopore 0.45-µm polycarbonate filters.

### Calculation of nutrient export and statistical methods

The annual Finnish riverine material export reported to HELCOM was calculated by utilising observed daily flow values and either monthly mean concentrations or estimated daily concentrations. In the former method, the monthly mean concentrations were multiplied by monthly sums of daily river discharges and the annual loads were summed from monthly loads (HELCOM 2011). In the latter one, the temporally nearest concentration observation was multiplied by the discharge observation of each day (periodic method, e.g. Kauppila and Koskiaho 2003).

The annual export figures calculated by the two different methods were comparable. In this article, we report only the results of the periodic method, because it was found to have the highest general reliability (lowest root-mean-squared error, RMSE) for the estimation of TN load (Kauppila and Koskiaho 2003).

The total nutrient export from unmonitored catchments (11% of the total catchment area) was estimated from nearby monitored catchments with similar land cover characteristics using an area-specific export coefficient (kg N or P per km^2^ catchment area).

The nutrient export was flow-normalised with a semiparametric method (Grimvall 2004) and with a method developed by Larsen and Svendsen (2013) for the use of HELCOM’s Pollution Load Compilation (PLC) data. The main difference between the normalisation methods is that the semiparametric model directly takes into account the seasonality of the flows and nutrient loads in the model parameters. The seasonality was also included in the calculations of the HELCOM PLC model by applying it for each month over the years. Overall, the flow normalisation methods gave comparable results, with only one exception in a relatively small river in the BOB sub-region. We chose to use the HELCOM method due to its simplicity and official status in HELCOM PLC.

Trends in export and concentration were analysed with the Mann–Kendall and seasonal Kendall tests (Hirsch et al. 1982, 1991) for annual and monthly export and concentration, respectively, using both non-adjusted as well as flow-adjusted values. If the test statistics were greater or lesser than zero on the 95% significance level, we detected an ‘upward trend’ or a ‘downward trend’, respectively. The magnitude of the trend was determined by the Theil–Sen slope estimator (Hirsch et al. 1982). The total change over the whole time series was calculated by multiplying the slope with the number of years minus one in the time series. The changes in point source loads were estimated by comparing the loads in 1995 and 2016.

## Results

### Trends in direct point source loads, riverine concentrations and export

TN inputs discharged directly to the sea from point sources started to decrease in the mid-1990s and from 1995 to 2016 they decreased by 3756 t (38%) (Figs. S1, 2a). In 2016, direct point source TN load was 6100 t, of which 65% originated from municipal WWTPs, 27% from industrial WWTPs and 8% from fish farms. The major reduction in direct TP inputs from point sources happened before 1995 and from 1995 to 2016 they further decreased by 196 t (54%) (Figs. S2, 2b). This was especially due to decreased loading from the pulp and paper industry and fish farms. In 2016, direct point source TP load was 167 t, of which 33% originated from municipal WWTPs, 42% from industrial WWTPs and 25% from fish farms.

**Fig. 2:**
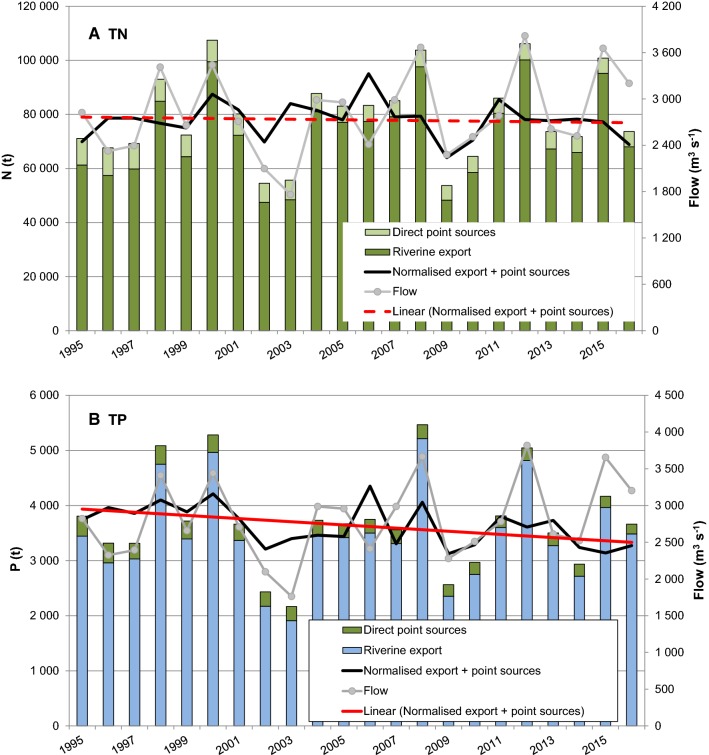
**a** TN inputs from Finland into the Baltic Sea in 1995–2016 and **b** TP inputs from Finland into the Baltic Sea in 1995–2016. Solid red line shows statistically significant trend and the respective dashed line statistically non-significant trend

In 1995–2016, upward trends of TN concentrations occurred in five rivers and a downward trend in three rivers (Table S3). NH_4_-N concentrations had downward trends in most of the rivers, whereas NO_2,3_-N concentrations had both upward and downward trends. Upward trends of both TP and PO_4_-P concentrations were detected in two rivers with intensive farming in the catchments, and downward trends of TP were found in six rivers, of which three were large rivers in southern Finland. Downward trends of TSS concentrations were discovered in six rivers and an upward trend in one river (Table S3).

**Fig. 3:**
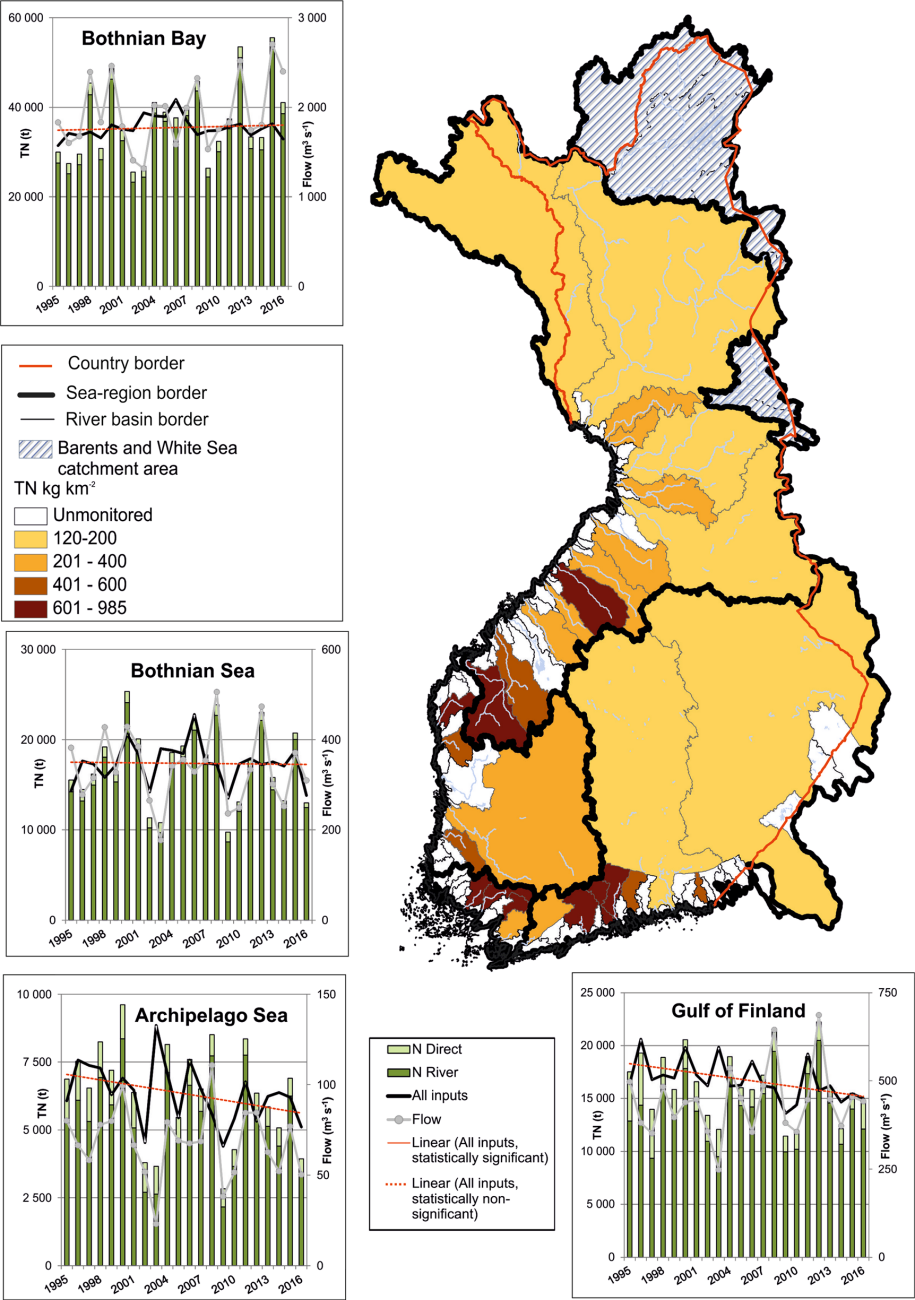
TN inputs from Finland into the Baltic Sea in 1995–2016 by sub-regions. Dark green bars show non-normalised export, light green bars direct point sources, grey line flow, black line flow-normalised total inputs. Solid red line shows statistically significant trend and the respective dashed line statistically non-significant trend. The map shows the area-specific (kg km^−2^) TN export of monitored rivers

The riverine export varied greatly between the years depending on changes in water flow, which in turn reflects the precipitation. The flow-normalised export also varied quite widely (Fig. 2a, b). There was no statistically significant trend in the flow or in the total riverine TN export (non-normalised or normalised) in 1995–2016. On the contrary, the sub-region-wise results indicated that there was an increasing tendency in the non-normalised TN export to the BOB, even though the increase was not statistically significant (Fig. 3). The river-wise examination of the TN export verified this, since the non-normalised export increased in four rivers in the BOB catchment (Table 1). The River Oulujoki in the BOB catchment was the only river in which there was a statistically significant upward trend in the flow-normalised TN export. The area-specific TN export varied from 120 to 985 kg km^−2^ between the river basins (Fig. 3). The highest values were observed in the southern and western catchments flowing through cultivated areas.

**Table 1:** Non-normalised and flow-normalised TN export in 1995 and trend statistics in 1995−2016. Downward arrow indicates statistically significant (*p* < 0.05) increase and upward arrow statistically significant decrease. Only statistically significant proportional (%) changes and trends are shown.

TN (1995–2016)			Non-normalised export						Flow-normalised export					
Basin no.	Sea region	River	Export in 1995 (t)	Slope (t a^−1^)	*p*	Change (t)	% Change	Trend	Export in 1995 (t)	Slope (t a^−1^)	*p*	Change (t)	% Change	Trend
4	GUF	VUOKSI	7879	89	0.135	1878			8263	6.1	0.382	128		
11	GUF	VIROJOKI	235	− 2	0.248	− 50			209	− 2.5	0.052	− 53		
14	GUF	KYMIJOKI	5530	41	0.297	862			6324	− 1.1	0.844	− 22		
16	GUF	KOSKENKYLÄNJOKI	361	7	0.185	154			428	3.0	0.248	63		
18	GUF	PORVOONJOKI	1136	− 6	0.592	− 129			1224	− 8.6	0.167	− 181		
19	GUF	MUSTIJOKI	680	– 3	0.756	− 71			656	− 0.9	0.844	− 19		
21	GUF	VANTAANJOKI	1394	− 5	0.672	− 112			1397	− 8.0	0.382	− 168		
23	GUF	KARJAANJOKI	556	− 4	0.382	− 83			518	0.9	0.672	18		
24	ARC	KISKONJOKI	210	2	0.446	51			228	0.1	0.888	3		
25	ARC	USKELANJOKI	578	− 8	0.150	− 172			501	− 3.1	0.446	− 66		
27	ARC	PAIMIONJOKI	969	− 11	0.271	− 235			905	− 4.8	0.517	− 100		
28	ARC	AURAJOKI	666	− 3	0.714	− 62			631	− 0.8	0.888	− 17		
34	BOS	EURAJOKI	623	− 3	0.714	− 59			705	− 1.0	0.844	− 22		
35	BOS	KOKEMÄENJOKI	10220	− 58	0.592	− 1209			9857	5.6	0.888	118		
37	BOS	LAPVÄÄRTINJOKI	511	1	0.592	28			508	− 1.0	0.554	− 22		
39	BOS	NÄRPIÖNJOKI	570	17	0.135	354			633	2.3	0.352	49		
42	BOB	KYRÖNJOKI	2699	67	0.059	1415			3416	14.3	0.382	301		
44	BOB	LAPUANJOKI	1823	44	0.135	916			2091	13.6	0.248	286		
49	BOB	PERHONJOKI	733	18	0.034	372	51	↗	843	1.8	0.672	39		
51	BOB	LESTIJOKI	390	6	0.204	126			487	− 4.1	0.108	− 86		
53	BOB	KALAJOKI	1783	54	0.076	1131			2332	− 5.7	0.592	− 120		
54	BOB	PYHÄJOKI	1085	17	0.225	363			1261	− 3.4	0.592	− 71		
57	BOB	SIIKAJOKI	1061	37	0.045	787	74	↗	1458	2.2	0.714	47		
59	BOB	OULUJOKI	2464	55	0.040	1158	47	↗	2865	27.8	0.004	584	20	↗
60	BOB	KIIMINGINJOKI	614	23	0.009	489	80	↗	827	− 2.4	0.414	− 50		
61	BOB	IIJOKI	1997	19	0.108	391			2272	1.3	0.844	28		
63	BOB	KUIVAJOKI	326	3	0.324	65			389	− 1.5	0.352	− 31		
64	BOB	SIMOJOKI	630	11	0.324	223			709	− 2.1	0.085	− 44		
65	BOB	KEMIJOKI	6011	27	0.592	576			6357	12.4	0.382	261		
67	BOB	TORNIONJOKI	3948	34	0.352	721			3890	1.4	0.714	29		

Trend: ↘ Decrease No change ↗ Increase

**Fig. 4:**
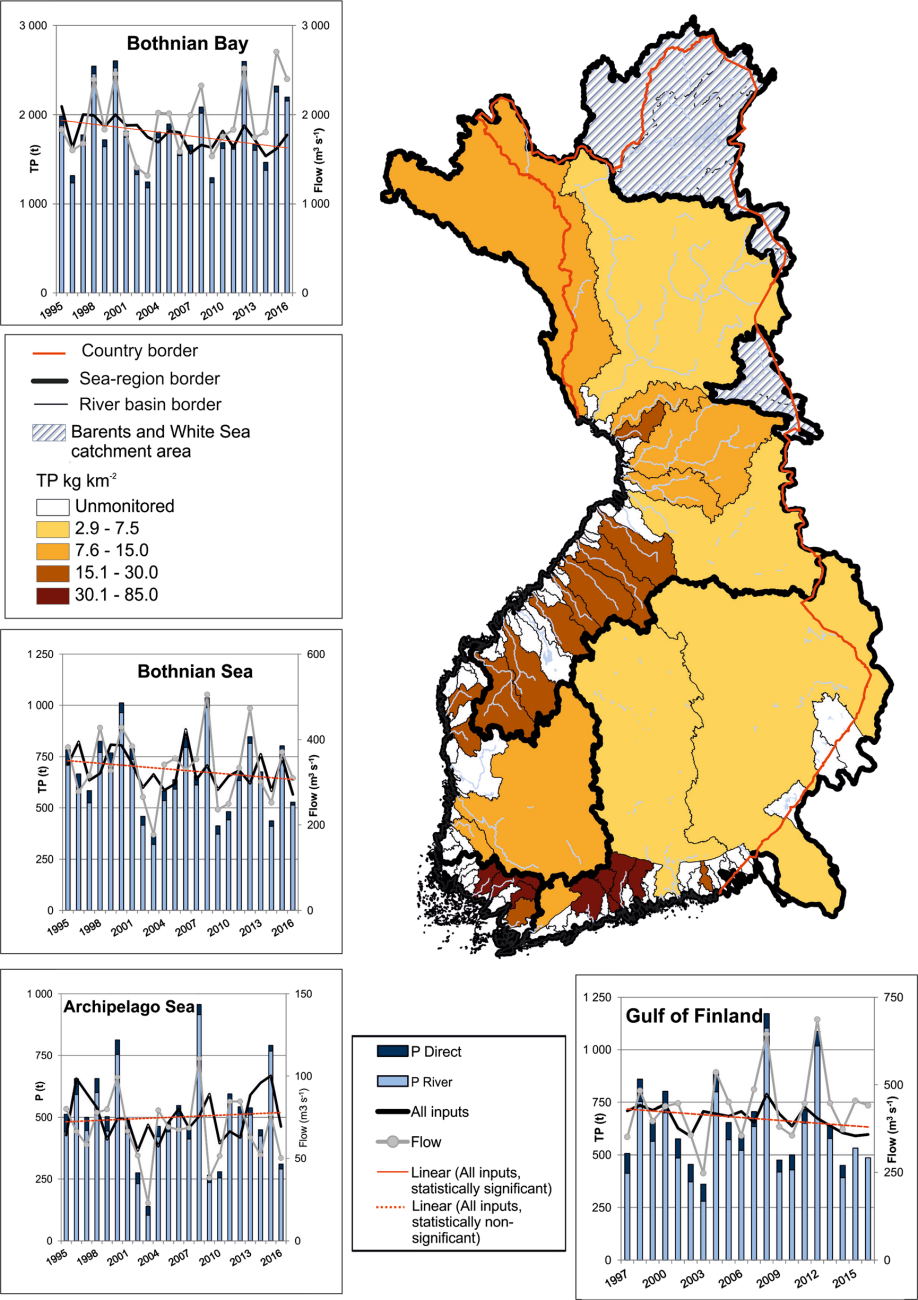
TP inputs from Finland into the Baltic Sea in 1995–2016 by sub-regions. Dark blue bars show non-normalised export, light blue bars direct point sources, grey line flow, black line flow-normalised total input. Solid red line shows statistically significant trend and the respective dashed line statistically non-significant trend. The map shows the area-specific (kg km^−2^) TP export of monitored rivers

The total Finnish TP inputs (direct point sources + riverine export) into the Baltic Sea decreased in 1995–2016 (Fig. 2b), but there was no statistically significant trend in the non-normalised TP export. BOB was the only sea-region showing a decrease: the flow-normalised TP export decreased by 19% (Fig. 4). Three upward trends were detected in the river-wise non-normalised TP export, whereas seven downward and two upward trends were found in the flow-normalised export (Table 2). The area-specific TP export varied from 3 to 85 kg km^−2^ between the river basins and the highest values were observed in the intensively cultivated ARC catchment (Fig. 4).

**Table 2:** Non-normalised and flow-normalised TP export in 1995 and trend statistics in 1995−2016. Downward arrow indicates statistically significant (*p* < 0.05) increase and upward arrow statistically significant decrease. Only statistically significant changes proportional (%) and trends are shown.

TP (1995–2016)			Non-normalised export						Flow-normalised export					
Basin no.	Sea region	River	Export in 1995 (t)	Slope (t a^−1^)	*p*	Change (t)	% Change	Trend	Export in 1995 (t)	Slope (t a^−1^)	*p*	Change (t)	% Change	Trend
4	GUF	VUOKSI	146	0.41	0.481	9			158	− 0.71	0.108	− 15		
11	GUF	VIROJOKI	7.6	− 0.02	0.714	0			8.4	− 0.06	0.076	− 1		
14	GUF	KYMIJOKI	195	− 1.14	0.554	− 24			235	− 2.43	0.017	− 51	− 22	↘
16	GUF	KOSKENKYLÄNJOKI	17.7	0.74	0.045	16	88	↗	23.4	0.42	0.003	9	37	↗
18	GUF	PORVOONJOKI	44.5	0.14	0.800	3			51.7	0.01	0.888	0		
19	GUF	MUSTIJOKI	35.5	− 0.61	0.248	− 13			30.8	− 0.13	0.352	− 3		
21	GUF	VANTAANJOKI	68.6	− 0.59	0.517	− 12			74.0	− 0.73	0.045	− 15	− 21	↘
23	GUF	KARJAANJOKI	20.1	− 0.15	0.297	− 3			17.1	0.08	0.135	2		
24	ARC	KISKONJOKI	11.6	0.09	0.352	2			11.8	0.08	0.135	2		
25	ARC	USKELANJOKI	49.1	− 0.33	0.714	− 7			41.3	0.41	0.414	9		
27	ARC	PAIMIONJOKI	68.7	− 0.09	0.978	− 2			62.6	0.88	0.248	18		
28	ARC	AURAJOKI	57.4	− 0.16	0.844	− 3			48.9	0.58	0.248	12		
34	BOS	EURAJOKI	21.9	− 0.41	0.150	− 9			23.7	− 0.36	0.096	− 8		
35	BOS	KOKEMÄENJOKI	401	− 5.10	0.248	− 107			396	− 2.77	0.167	− 58		
37	BOS	LAPVÄÄRTINJOKI	31.8	− 0.16	0.672	− 3			29.6	− 0.14	0.632	− 3		
39	BOS	NÄRPIÖNJOKI	16.8	0.88	0.019	18	110	↗	17.5	0.54	0.002	11	64	↗
42	BOB	KYRÖNJOKI	117	0.75	0.481	16			130	− 0.48	0.554	− 10		
44	BOB	LAPUANJOKI	74.1	0.43	0.592	9			75.9	− 0.18	0.481	− 4		
49	BOB	PERHONJOKI	42.1	0.34	0.517	7			52.0	− 0.65	0.002	− 14	− 26	↘
51	BOB	LESTIJOKI	33.4	− 0.48	0.324	− 10			36.9	− 0.83	0.001	− 17	− 47	↘
53	BOB	KALAJOKI	129	− 1.22	0.297	− 26			48	− 2.98	0.000	− 63	− 42	↘
54	BOB	PYHÄJOKI	55.5	0.50	0.297	10			68.6	− 0.43	0.085	− 9		
57	BOB	SIIKAJOKI	75.9	1.04	0.324	22			107.4	− 0.75	0.030	− 16	− 15	↘
59	BOB	OULUJOKI	127	0.94	0.481	20			144	− 0.01	0.844	0		
60	BOB	KIIMINGINJOKI	38.4	0.86	0.045	18	47	↗	47.9	− 0.16	0.554	− 3		
61	BOB	IIJOKI	127	0.42	0.632	9			142	− 0.78	0.085	− 16		
63	BOB	KUIVAJOKI	16.8	0.23	0.324	5			20.4	− 0.03	0.632	− 1		
64	BOB	SIMOJOKI	27.9	0.26	0.592	6			31.1	− 0.07	0.446	− 2		
65	BOB	KEMIJOKI	347	− 2.32	0.672	− 49			380	− 4.27	0.040	− 90	− 24	↘
67	BOB	TORNIONJOKI	244	2.52	0.481	53			270	− 0.22	0.844	− 5		

Trend: ↘ Decrease ↔ No change ↗ Increase

The trends in TN concentrations and non-normalised TN export correlated negatively with the proportional (%) area of cultivated fields and urban areas (Table 3), whereas they correlated positively with the proportional area of peatlands and ditched peat area. The trends in flow-normalised TP export correlated positively with cultivated areas and negatively with ditched areas.

**Table 3:** Pearson correlation coefficient (*r*) with *p* value between proportional (%) catchment characteristics and trends of riverine concentrations and export. Statistically significant *r* on 5% significant level in bold. degrees of freedom = 28.

	Water		Forests		Cultivated areas		Urban areas		Peatlands		Ditched areas	
	*r*	*p*	*r*	*p*	*r*	*p*	*r*	*p*	*r*	*p*	*r*	*p*
Concentration												
TN	0.072	0.705	0.159	0.402	**− 0.427**	**0.019**	**− 0.531**	**0.003**	**0.523**	**0.003**	**0.560**	**0.001**
NO_2,3_-N	0.065	0.733	0.034	0.857	0.084	0.661	− 0.035	0.853	− 0.039	0.837	0.155	0.414
TP	− 0.279	0.135	0.040	0.836	0.092	0.630	− 0.090	0.635	− 0.002	0.991	− 0.057	0.765
SS	− 0.078	0.684	0.223	0.236	− 0.023	0.905	− 0.126	0.507	− 0.133	0.484	− 0.222	0.238
Export												
TN	− 0.087	0.647	0.090	0.636	**− 0.475**	**0.008**	**− 0.542**	**0.002**	**0.642**	**0.000**	**0.727**	**0.000**
TN norm.	0.229	0.224	0.071	0.708	− 0.176	0.353	− 0.229	0.223	0.097	0.611	0.105	0.582
TP	− 0.230	0.222	0.046	0.809	− 0.120	0.529	− 0.272	0.146	0.258	0.169	0.239	0.203
TP norm.	− 0.242	0.198	− 0.177	0.349	**0.468**	**0.009**	0.217	0.248	− 0.333	0.073	**− 0.366**	**0.047**

### Seasonal shifts in nutrient export

**Fig. 5:**
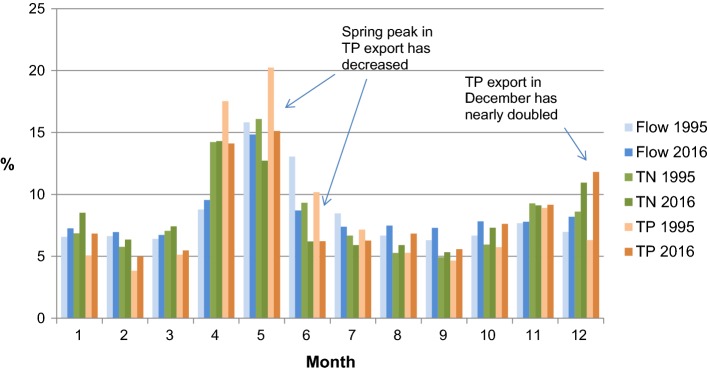
Flow and exports of TN and TP as monthly proportions (%) of annual totals in 1995 and 2016

The major part of nutrient export happens during spring thaw, which usually starts in April in southern Finland and in May–June in northern parts of the country. During the two past decades spring thaw has started earlier and its water volume has decreased, which on the other hand has been compensated by the increased flow during other months (Fig. 5). These changes, together with increased air temperature, have caused shifts in seasonal nutrient export: Nutrient export during spring thaw has decreased and increased during the winter months. The shifts were more evident in export, particularly in TP export, than the respective shifts in flow.

One distinct feature in the nutrient export during the last two decades in the southern Finnish rivers, especially those flowing through intensively cultivated areas, was that in mild and rainy winters (e.g. 2015) more than half of the annual TP export occurred in December (from mainly unfrozen soils, Fig. S3). The increase in the respective TN export was much weaker.

## Discussion

### Trends in water flow, riverine nutrient concentrations and export

Both seasonal and annual fluctuation in flow affects the amount of nutrient losses and subsequently the analysis of trends in nutrient losses (Stålnacke et al. 2014). The basic idea of flow normalisation is to detect trends unaffected by changes in flow, which enables, e.g., the evaluation of the effectiveness of water protection measures. In Finnish rivers, TP concentrations correlate more closely with flow than TN concentrations (TP median *r* 0.46, TN median *r* 0.32; Table S2), and therefore the variation in TP export caused by flow can be more effectively eliminated. Positive correlation between nutrient concentrations and flow is usually strong if most of the nutrient inputs originate from diffuse sources sensitive to changes in precipitation, such as cultivated areas and storm waters. In contrast, this correlation is weak or negative if a river receives a large amount of nutrient pollution originating from point sources (basins 14, 18, 21, 23, 35; Table S2). The correlation between nutrient concentrations and flow was low in river catchments with a high lake percentage due to the retention of nutrients in the lakes (basins 4, 14, 23, 35, 59; Tables S1, S2).

Concentrations of NH_4_-N decreased in most of the rivers, indicating that the overall water quality has improved in Finnish rivers due to improved municipal and industrial wastewater treatment, including nitrification of ammonium. This was partly reflected in the increased NO_2,3_-N concentrations since the NH_4_-N concentrations decreased in five rivers simultaneously as the NO_2,3_-N concentrations increased without any change in the TN concentration. However, since inorganic N (NH_4_-N + NO_2,3_-N) concentrations had a commonly downward trend in 1995–2016 (Table S3), we could attribute the increases in TN concentrations largely to increased organic N concentrations. This is also supported by the increasing TOC concentrations detected in many Finnish rivers (Räike et al. 2016), since organic N is usually bound to humic substances in boreal rivers.

The nutrient reduction targets are based on flow-normalised loads. It is important to notice that there was a clear difference between non-normalised and flow-normalised TP export trends: The non-normalised TP export did not show any decrease, whereas the flow-normalised TP export commonly decreased (Table 2). Thus, there is no indication that the actual (non-normalised) TP export from Finnish rivers would decrease in near the future and if the estimation of reduction targets would be based on the actual loads, Finland would face even more severe challenges in reaching the reduction targets. The discrepancy between non-normalised and flow-normalised TP export trends may be related to mild, rainy winters and shifts in the seasonality of flow (e.g. flow-normalisation does not take into account the effect of temperature on leaching of nutrients). It can also be partly rationalised by decreasing TP concentrations in several rivers: due to mitigation measures TP concentrations have decreased, but several factors connected to climate/weather (e.g. temperature and precipitation) have counteracted these mitigation measures targeted to reduce nutrient export into the Baltic Sea.

One distinct difference compared to the trends in the Finnish riverine phosphorus concentrations in 1975–2000 (Räike et al. 2003) was that the TP concentrations did not continue to decrease anymore in 1995–2016 in many rivers previously heavily polluted by point source loading. This indicates that the diffuse loading has not remarkably, if at all, decreased during the last 20 years since major reductions in the point source TP loading occurred before the turn of the century. Our results are in line with a recent study by Oelsner and Stets (2019) who found that decreasing TP yields in the conterminous US rivers were common among urban sites, but increases in TP loads could be detected in agricultural sites in 2002–2012. Also in southwestern European rivers decreases in P export were linked to decreased P loading from point sources, whereas N export did not show any clear trends (Romero et al. 2013).

Finnish agricultural water protection measures have especially been targeted to prevent the erosion of agricultural land, with the adoption of the EU’s Agri-Environment Programme projected to decrease erosion by 20–40% (Valpasvuo-Jaatinen et al. 1997). If we evaluate the success of erosion control on the basis of the trends in TSS, the results are meagre: TSS export decreased only in one river draining the most intensively cultivated areas (data not shown).

### Driving forces behind the changes

The nutrient loads originating from point sources have been substantially (TN 38% and TP 54%) reduced since 1995, and their proportion of the total inputs (point sources + riverine export) is nowadays less than 15%. Unlike in some other Baltic Sea countries, e.g. Russia, further declines in riverine nutrient export can still be achieved through modernisation of WWTPs (Knuuttila et al. 2017), but in Finland it is presently not possible to substantially increase nutrient removal from point sources. Therefore, the main question remains how to reduce diffuse loads, especially those originating from agriculture.

The average fertilisation rates in Finland have decreased from 40 to 8 kg P ha^−1^ in 1995–2010 (Tattari et al. 2017), but the decrease in the TP concentrations or in the flow-normalised export did not occur in the most intensive agricultural catchments in the ARC region. The relationships between the P supply and P concentration in the water column and ecological response are complex, and the capacity of riverine ecosystems to assimilate P is spatially and temporally very variable (Withers and Jarvie 2008). The time lag between the reduced fertilisation and decreased concentrations in water bodies is often fairly long, because accumulated P may continue to mobilise long after inputs decline (Powers et al. 2016). This accumulation is called legacy P (McCrackin et al. 2018).

Our results are in line with Ekholm et al. (2015) who found that the agricultural load of TN increased, especially in the rivers discharging into the BOB. One possible explanation is that even if the total area of croplands has been quite stable in Finland since 1990, the share of organic soils has increased by 42 900 ha in 1990–2016, predominantly in the catchment area of BOB. Animal production and farm enlargement are more common in the eastern and northern parts of the country where the occurrence of peat soils is also high (Kekkonen et al. 2019). Cleared new fields on organic soil types have been shown to have four times higher specific TN loading value than fields on mineral soil, while land clearing was not observed to have any effect on the TP load (Rankinen et al. 2016).

The TN export into BOB also increased in rivers with catchments not predominantly covered by cultivated areas and we could link the increases to ditching of peatlands. Peatlands cover one-third of the Finnish land area and about half of them have been ditched predominantly for forestry and to a lesser degree for peat mining purposes. The most intensive drained peatlands locate in the BOB catchment. Drainage activity peaked in 1970 and thereafter the emphasis turned to the maintenance of existing ditch networks (Joensuu et al. 2002). Tattari et al. (2017) found that the TN concentrations in most Finnish streams draining monitored forested areas increased in 1987–2011, whereas the TP concentrations decreased, probably due to the reduced forest TP fertilisation. In a recent study, Nieminen et al. (2017) concluded that the forestry-drained peatlands may contribute considerably more to nutrient load of watercourses than was previously estimated.

Algae need macronutrients in a certain ratio, and focusing on strictly P has led to excess N concentrations in relation to P in many Finnish freshwater systems. (The average TN:TP ratio in Finnish riverine export in 1975–1979 was 16.5, whereas in 2012–2016 it was 21.8.) The N:P ratio in freshwater bodies has also increased globally (Beusen et al. 2016). This may have partly increased TN export to the coastal waters as N in lakes is in excess in regard to algal production, and it is therefore retained to a lesser extent in the watercourses (Stålnacke et al. 1999). In Finland, increased TN export occurred, in the lake-rich catchment of the River Oulujoki, but also in lake-poor catchments, indicating that the reduced retention was not the major driver behind increases in the riverine TN export.

### Nutrient export in a warming climate

Mild winters have become more common in Finland and the annual precipitation has increased in the last 100 years (Irannezhad et al. 2014). The annual mean temperature is projected to rise by 2–5 °C and the annual mean precipitation by 0–30% by the 2050s, relative to the baseline period 1961–1990. Also the intensity of rainfall events is likely to enhance the contribution of high flow events to the annual loads (Ockenden et al. 2016). The projected precipitation changes are largest in winter and smallest in summer (Jylhä et al. 2004).

Snow cover will diminish or almost vanish in southern Finland, and its duration will become shorter (Heino et al. 2008). The relative importance of the spring snowmelt in material export will decrease in wet and warm years (Mattsson et al. 2015). Based on our results, since the beginning of this century more than half of the annual TP export frequently happened within a couple of weeks in December–January in rivers flowing through agricultural areas in southern Finland (Fig. S3), and during those years the total annual export was above the long-term average. In the UK in two small catchments 80% of the TP export was detected to happen during the highest discharge events (Ockenden et al. 2016). Unfrozen soil, with thin or no snow cover, increases erosion and the leaching of nutrients to surface waters. Soil temperature is a major factor affecting organic matter decomposition, and climate change is assumed to accelerate N mineralisation and thus increase N concentrations and leaching in both agricultural and forested soils (Tattari et al. 2017).

If the tendency of mild winters continues, nutrient loads from agriculture are projected to increase in the future (Huttunen et al. 2015), assuming that the water protection measures cannot reduce the load from the current level.

### Reaching the nutrient reduction targets and effectiveness of water protection measures

Finland reduced its annual TP inputs by only 87 t from 1995 to 2016 and there was no statistically significant decrease in the TN inputs; thus, there has not been much progress in reaching the original BSAP reduction targets (3030 t TN and 356 t TP). Finland’s national nutrient reduction targets for coastal waters required by WFD’s RBMP are higher for both TP and TN compared to the BSAP targets for the open sea area of the Baltic Sea. (The only exception compared with the BSAP is the P target for the GUF.) Even if all the existing measures included in WFD and the new measures listed in the MSFD programme were fully implemented, none of the sea regions will achieve RBMP reduction targets by 2020.

Agriculture was already identified as the largest source of TP and TN in surface waters over 30 years ago (Kauppi 1984), but we still but we still do not see any significant results achieved by water protection measures in reducing load originating from cultivated areas in Finland. The Finnish Agri-Environmental Programme (FAEP), launched in 1995, forms the most important policy instrument for controlling agricultural nutrient loading, but the lack of improvements in water quality despite a large number of water protection measures taken has been demonstrated also in earlier studies (e.g. Granlund et al. 2005). Currently, there is a strong national impetus in Finland to enhance the recycling of manure-based nutrients in the spirit of a circular economy, but so far this recycling has not been efficient due to technical, economic and regulatory hindrances. The annually produced manure TP would suffice to satisfy the needs of plants in the whole cultivated area of Finland without any need for commercial fertilisers, assuming that manure can be transported to the fields in need of P addition (Ylivainio et al. 2014).

Problems in reducing nutrient loading from areas impacted by intensive anthropogenic activities causing so-called effective biogeochemical stationarity have also been evident globally (Basu et al. 2010; McCrackin et al. 2014; Stålnacke et al. 2014; Van Meter et al. 2017). Presently P loads into the Baltic Sea are dominated by mobile legacy sources and the there is a need for a long-term perspective in eutrophication management (McCrackin et al. 2018).

## Conclusions

Finnish point source TN and TP loads into the Baltic Sea have decreased substantially during the last two decades, but loads originating from diffuse sources remain a huge and possibly increasing challenge. There have been no signs of decrease in riverine TN export; on the contrary, it is on the rise in the BOB sub-region. The likely reasons for this trend are accelerated mineralisation of organic matter in a warming climate, forestry practices, changes in hydrology and increased cultivation on organic soils. The flow-normalised riverine P export decreased in many rivers, but the non-normalised export did not decrease in any of the rivers, indicating that the actual export has remained more or less at the same level since 1995. The discrepancy between the non-normalised and flow-normalised TP export was partly rationalised by decreasing TP concentrations in several rivers: Due to mitigation measures TP concentrations have decreased, but several factors connected to climate/weather (e.g. temperature and precipitation) have counteracted these mitigation measures targeted to reduce nutrient export into the Baltic Sea. In 2016 Finland was far from reaching the nutrient reduction targets of HELCOM’s BSAP or the national WFD’s RBMPs.

## Electronic supplementary material

Below is the link to the electronic supplementary material.

Supplementary material 1 (PDF 292 kb)
