# Supplementary material for: Nutrient export from Finnish rivers into the Baltic Sea has not decreased despite water protection measures
Source: Ambio. 2019 Jul 5;49(2):460–74. doi: 10.1007/s13280-019-01217-7 (PMC6965342; doi:10.1007/s13280-019-01217-7)
Supplement: Supplementary file 1 — Supplementary material 1 (PDF 292 kb) [file 13280_2019_1217_MOESM1_ESM.pdf]

Ambio

Electronic Supplementary Material
This supplementary material has not been peer reviewed.

Title: Nutrient export from Finnish rivers into the Baltic Sea has not decreased despite water protection measures

Authors: Antti Räike, Antti Taskinen, Seppo Knuuttila

Table S1. Land cover characteristics and runoff.

Basin number	River	Area km^2	Runoff mm	Water %	Field %	Forest %	Urban %	Peatland %	Open %	Peatland[1)] %	Ditched %
4	VUOKSI	61466	319	19	5	45	3	14	13	19	15
11	VIROJOKI	357	392	3	15	54	4	10	15	15	13
14	KYMIJOKI	37159	262	18	7	49	4	8	13	13	11
16	KOSKENKYLÄNJOKI	895	287	4	30	40	6	3	17	5	4
18	PORVOONJOKI	1273	320	2	31	38	10	3	17	5	3
19	MUSTIJOKI	783	283	2	30	39	8	6	15	8	6
21	VANTAANJOKI	1686	303	2	25	37	20	4	13	7	5
23	KARJAANJOKI	2046	289	11	18	46	9	3	12	6	5
24	KISKONJOKI	1047	312	6	23	48	7	5	12	8	6
25	USKELANJOKI	566	328	1	43	34	9	4	10	6	5
27	PAIMIONJOKI	1088	276	2	43	34	8	4	10	7	5
28	AURAJOKI	874	288	1	37	33	12	7	11	10	6
34	EURAJOKI	1336	236	13	23	39	7	8	10	12	9
35	KOKEMÄENJOKI	27046	281	11	16	46	6	9	12	13	10
37	LAPVÄÄRTINJOKI	1098	409	0	13	50	3	22	11	32	25
39	NÄRPIÖNJOKI	992	291	1	21	47	4	16	11	23	17
42	KYRÖNJOKI	4923	275	1	25	36	5	19	13	24	18
44	LAPUANJOKI	4122	245	3	22	39	5	18	14	25	20
49	PERHONJOKI	2524	300	3	10	38	3	30	16	38	27
51	LESTIJOKI	1373	283	6	11	39	2	25	17	36	26
53	KALAJOKI	4247	306	2	16	47	3	17	15	31	26
54	PYHÄJOKI	3712	276	5	10	50	3	18	13	35	30
57	SIIKAJOKI	4218	310	2	9	45	2	32	10	52	41
59	OULUJOKI	22845	359	12	3	44	2	23	17	36	26
60	KIIMINGINJOKI	3814	367	3	2	39	2	40	14	53	31
61	IIJOKI	14191	375	6	2	42	1	31	18	42	24
63	KUIVAJOKI	1355	388	3	2	40	1	40	15	57	29
64	SIMOJOKI	3160	427	6	2	37	1	39	16	52	29
65	KEMIJOKI	51127	348	4	1	52	1	24	18	37	10
67	TORNIONJOKI	34781	381	5	1	49	1	19	25	34	12

[1)] Source: Geological Survey of Finland

Table S2. Pearson correlation (r) flow vs. TN & TP concentrations and the number of annual TN and TP analyses from 1995 to 2016,

Basin number	River	Parameter	Flow vs. concentration r	Number of annual samples 1995	1996	1997	1998	1999	2000	2001	2002	2003	2004	2005	2006	2007	2008	2009	2010	2011	2012	2013	2014	2015	2016
4	VUOKSI	TN	0.010	32	36	33	25	23	25	26	24	24	23	24	24	24	27	27	27	22	23	28	30	22	12
11	VIROJOKI	TN	0.534	6	6	7	15	18	16	15	14	20	11	17	12	12	11	11	12	11	12	10	10	9	13
14	KYMIJOKI	TN	0.037	27	32	35	27	28	30	26	26	25	23	30	26	23	24	23	23	32	22	21	21	16	13
16	KOSKENKYLÄNJOKI	TN	0.017	17	17	27	23	28	29	24	24	21	24	27	28	23	27	22	21	20	26	22	22	21	19
18	PORVOONJOKI	TN	-0.099	14	12	24	21	25	26	21	24	21	25	27	28	23	30	25	24	23	26	22	22	21	19
19	MUSTIJOKI	TN	0.293	14	13	24	20	25	26	21	24	21	25	27	28	23	27	22	21	20	25	26	22	21	19
21	VANTAANJOKI	TN	0.292	14	12	24	20	25	29	22	24	21	25	27	29	22	31	25	24	23	26	22	22	21	19
23	KARJAANJOKI	TN	0.083	19	17	27	26	22	31	22	26	26	25	25	28	30	27	25	22	20	23	21	27	20	13
24	KISKONJOKI	TN	0.438	12	13	12	16	13	21	22	21	20	22	20	24	22	22	25	22	21	21	17	21	20	13
25	USKELANJOKI	TN	0.328	15	16	16	15	17	24	25	23	25	23	21	24	22	22	20	22	21	22	23	23	19	19
27	PAIMIONJOKI	TN	0.138	15	15	15	15	17	20	25	22	24	23	23	26	24	25	27	24	24	21	22	22	19	20
28	AURAJOKI	TN	0.218	28	30	28	35	25	43	39	28	33	27	30	39	43	42	56	42	49	49	44	37	34	24
34	EURAJOKI	TN	0.470	18	21	16	21	20	21	20	17	21	17	19	21	19	20	19	19	17	25	24	19	18	14
35	KOKEMÄENJOKI	TN	0.376	18	21	19	20	20	19	19	20	21	19	15	19	18	20	19	19	19	24	20	23	21	15
37	LAPVÄÄRTINJOKI	TN	0.534	11	12	12	12	14	18	13	13	13	13	13	13	16	15	13	13	12	16	15	19	15	12
39	NÄRPIÖNJOKI	TN	0.314	11	11	14	10	16	16	13	17	15	16	16	16	17	15	15	16	15	17	13	16	16	18
42	KYRÖNJOKI	TN	0.515	23	14	15	17	13	17	14	16	17	14	14	19	17	14	14	15	18	19	16	17	15	17
44	LAPUANJOKI	TN	0.567	6	11	12	13	18	16	15	17	18	15	13	13	15	13	13	13	13	13	13	15	12	13
49	PERHONJOKI	TN	0.273	14	15	12	18	17	16	15	17	13	14	13	13	19	16	13	13	17	17	15	14	12	14
51	LESTIJOKI	TN	0.434	15	15	10	19	13	16	18	17	13	14	13	13	17	16	15	18	18	17	13	16	14	13
53	KALAJOKI	TN	0.486	16	15	12	15	13	21	15	14	14	15	14	18	19	15	17	16	16	13	13	13	13	13
54	PYHÄJOKI	TN	0.565	11	16	18	17	19	19	19	14	17	20	17	18	22	20	19	16	16	17	20	15	15	18
57	SIIKAJOKI	TN	0.609	18	22	25	22	23	23	25	20	23	19	16	18	18	20	17	13	15	13	14	14	13	13
59	OULUJOKI	TN	-0.114	14	22	21	20	22	22	20	20	21	14	14	14	15	16	13	13	13	16	18	13	12	13
60	KIIMINGINJOKI	TN	0.303	15	14	20	26	24	22	24	18	21	17	18	18	17	17	15	14	12	13	13	13	13	13
61	IIJOKI	TN	0.431	18	16	21	19	23	24	23	19	18	17	18	17	18	17	16	14	14	14	14	14	14	13
63	KUIVAJOKI	TN	-0.207	13	14	18	18	18	25	26	22	23	18	17	19	18	17	17	14	14	14	12	11	10	4
64	SIMOJOKI	TN	0.392	16	12	17	17	17	17	16	20	22	19	19	14	13	13	13	14	13	15	14	13	13	13
65	KEMIJOKI	TN	0.529	16	12	17	17	17	18	18	19	18	16	18	15	15	15	14	14	13	14	14	14	13	13
67	TORNIONJOKI	TN	0.241	15	12	17	17	17	17	16	18	18	17	18	20	21	22	20	17	17	18	18	18	17	17
4	VUOKSI	TP	-0.089	31	36	33	25	23	25	26	24	24	23	24	24	24	27	27	27	23	24	28	30	22	12
11	VIROJOKI	TP	0.434	6	6	7	16	19	16	15	14	20	11	17	12	12	11	11	12	11	12	10	10	9	13
14	KYMIJOKI	TP	-0.015	27	32	24	23	28	30	26	26	25	26	30	26	23	24	23	23	32	22	21	21	16	17
16	KOSKENKYLÄNJOKI	TP	0.076	17	17	27	23	28	29	24	24	21	24	27	28	23	27	22	21	20	26	22	22	21	19
18	PORVOONJOKI	TP	0.553	14	12	24	21	25	26	21	24	21	25	27	28	23	30	25	24	23	26	22	22	21	19
19	MUSTIJOKI	TP	0.614	14	13	24	20	25	26	21	24	21	25	27	28	23	27	22	21	20	25	26	22	21	19
21	VANTAANJOKI	TP	0.637	14	12	24	20	25	29	22	24	21	25	27	29	23	31	25	24	23	26	22	22	21	19
23	KARJAANJOKI	TP	0.081	19	17	27	26	22	31	22	26	26	25	25	28	30	26	25	22	20	23	21	27	20	13
24	KISKONJOKI	TP	0.068	11	12	11	16	13	22	22	21	21	22	20	24	22	22	25	22	21	21	17	21	20	13
25	USKELANJOKI	TP	0.647	15	16	16	15	17	25	25	23	25	23	21	24	22	22	20	22	21	22	23	23	19	19
27	PAIMIONJOKI	TP	0.369	15	15	15	15	17	21	25	22	24	24	23	26	24	25	27	25	24	21	22	22	19	20
28	AURAJOKI	TP	0.546	30	32	30	37	27	46	40	30	34	28	32	40	45	44	58	44	51	51	46	39	36	25
34	EURAJOKI	TP	0.476	18	20	16	21	20	20	20	17	21	17	19	21	19	20	19	19	17	25	24	19	18	14
35	KOKEMÄENJOKI	TP	0.475	18	21	19	20	20	18	19	19	21	19	15	19	18	20	18	19	19	24	20	23	21	15
37	LAPVÄÄRTINJOKI	TP	0.603	11	12	13	12	14	20	15	15	13	13	13	13	16	15	13	13	12	16	15	19	17	12
39	NÄRPIÖNJOKI	TP	0.178	11	11	15	10	16	16	13	17	15	16	16	16	17	15	15	16	15	17	13	16	16	18
42	KYRÖNJOKI	TP	0.710	23	14	15	17	13	17	14	16	17	14	14	19	17	14	14	15	18	19	16	17	15	17
44	LAPUANJOKI	TP	0.438	6	11	12	13	18	16	15	17	18	15	13	12	15	13	13	13	13	13	13	15	12	13
49	PERHONJOKI	TP	0.363	14	15	12	18	17	16	15	17	13	14	13	13	19	16	13	13	17	17	15	14	12	14
51	LESTIJOKI	TP	0.496	15	15	10	19	13	16	18	17	13	14	13	13	17	16	15	18	17	17	12	16	14	13
53	KALAJOKI	TP	0.606	16	15	12	15	13	21	15	14	15	16	13	18	19	15	16	16	15	13	13	13	13	13
54	PYHÄJOKI	TP	0.703	11	16	18	17	19	19	19	14	17	20	17	18	22	20	19	16	15	17	20	15	15	18
57	SIIKAJOKI	TP	0.437	18	22	25	22	23	23	25	20	23	19	16	18	18	20	17	13	14	13	14	15	13	13
59	OULUJOKI	TP	-0.173	14	21	21	20	22	22	20	20	21	14	14	14	15	16	13	13	12	16	18	13	12	13
60	KIIMINGINJOKI	TP	0.388	15	13	20	26	24	22	24	19	21	17	18	18	17	17	16	14	11	13	12	14	13	13
61	IIJOKI	TP	0.420	18	15	21	19	22	24	23	19	18	17	18	17	18	17	16	14	13	14	14	15	14	13
63	KUIVAJOKI	TP	-0.030	13	14	18	18	18	25	26	23	23	18	18	19	18	17	17	14	13	14	12	12	10	4
64	SIMOJOKI	TP	0.693	16	12	17	17	17	17	16	20	22	19	19	14	13	13	13	14	13	15	14	13	13	13
65	KEMIJOKI	TP	0.842	16	12	17	17	17	18	18	19	18	16	18	14	15	16	14	14	13	14	14	14	6	13
67	TORNIONJOKI	TP	0.534	15	12	17	17	17	17	16	18	18	17	18	19	21	22	20	17	17	18	18	18	10	17

Table S3. Trends in riverine TN, NH4-N, NO2,3-N, TP, PO4-P and suspended solids concentrations in 1995−2016. Downward arrow indicates statistically significant ($p<0.05$) decrease and upward arrow statistically significant increase. Only statistically significant slopes and trends are shown.

1995-2016		TN			NH_4-N			$NO_{2,3}$-N			TP			PO_4-P			Suspended solids		
River	Basin no	p	Slope	Trend	p	Slope	Trend	p	Slope	Trend	p	Slope	Trend	p	Slope	Trend	p	Slope	Trend
VUOKSI	4	0.320			0.003	-0.08	↘	0.973			0.014	-0.04	↘				0.308		
VIROJOKI	11	0.322			0.002	-0.75	↘	0.018	-5.00	↘	0.832			0.298	0.00		0.444		
KYMIJOKI	14	0.747			0.000	-0.88	↘	0.002	2.64	↗	0.002	-0.25	↘	0.216	0.00		0.211		
KOSKENKYLÄNJOKI	16	0.040	14.1	↗	0.190			0.595			0.002	1.84	↗	0.000	0.24	↗	0.009	1.14	↗
PORVOONJOKI	18	0.042	-35.0	↘	0.129			0.063			0.758			0.018	-0.31	↘	0.686		
MUSTIJOKI	19	0.954			0.003	-1.20	↘	0.335			0.814			0.052	-0.13		0.828		
VANTAANJOKI	21	0.017	-18.2	↘	0.017	-1.22	↘	0.041	-13.9	↘	0.363			0.022	-0.14	↘	0.338		
KARJAANJOKI	23	0.979			0.000	-3.17	↘	0.469			0.355			0.148	0.00		0.169		
KISKONJOKI	24	0.479			0.057			0.099			0.648			0.436	0.00		0.479		
USKELANJOKI	25	0.113			0.002	-2.27	↘	0.080			0.602			0.046	-0.29	↘	0.422		
PAIMIONJOKI	27	0.942			0.572			0.845			0.583			0.239	0.15		0.649		
AURAJOKI	28	0.675			0.799			0.747			0.386			0.162	-0.17		0.075		
EURAJOKI	34	0.971			0.021	-3.00	↘	0.456			0.054			0.127	0.00		0.010	-0.28	↘
KOKEMÄENJOKI	35	0.450			0.001	-1.86	↘	0.028	5.68	↗	0.001	-0.43	↘	0.001	-0.13	↘	0.008	-0.20	↘
LAPVÄÄRTINJOKI	37	0.399			0.000	-1.60	↘	0.633			0.109			0.059	-0.14		0.549		
NÄRPIÖNJOKI	39	0.003	22.7	↗	0.000	-6.00	↘	0.000	35.4	↗	0.001	1.89	↗	0.000	0.80	↗	0.414		
KYRÖNJOKI	42	0.069			0.001	-3.33	↘	0.028	17.5	↗	0.071			0.103	0.14		0.612		
LAPUANJOKI	44	0.643			0.000	-4.45	↘	0.040	8.22	↗	0.013	-0.60	↘	0.154	-0.11		0.633		
PERHONJOKI	49	0.265			0.001	-1.82	↘	0.340			0.148			0.930	0.00		0.015	-0.16	↘
LESTIJOKI	51	0.836			0.001	-1.13	↘	0.440			0.009	-0.59	↘	0.038	-0.20	↘	0.719		
KALAJOKI	53	0.212			0.002	-1.20	↘	0.402			0.001	-1.05	↘	0.117	-0.20		0.992		
PYHÄJOKI	54	0.156			0.028	-0.40	↘	0.589			0.925			0.984	0.00		0.012	-0.23	↘
SIIKAJOKI	57	0.022	9.5	↗	0.032	-0.53	↘	0.402			0.631			0.374	-0.08		0.047		
OULUJOKI	59	0.003	3.2	↗	0.001	-0.29	↘	0.045	0.58	↗	0.361			0.345	0.00		0.098		
KIIMINGINJOKI	60	0.044	2.5	↗	0.114			0.008	-0.99	↘	0.431			0.185	0.00		0.298		
IIJOKI	61	0.175			0.004	-0.17	↘	0.001	-0.80	↘	0.339			0.262	0.00		0.022	-0.08	↘
KUIVAJOKI	63	0.018	-4.4	↘	0.000	-0.57	↘	0.001	-3.23	↘	0.315			0.030	-0.14	↘	0.031	-0.23	↘
SIMOJOKI	64	0.452			0.043	-0.05	↘	0.006	-0.56	↘	0.363			0.888	0.00		0.536		
KEMIJOKI	65	0.521			0.063			0.435			0.099			0.482	0.00		0.633		
TORNIONJOKI	67	0.645			0.418			0.017	-0.11	↘	0.863			0.947	0.00		0.978		
Trend:	↘ Decrease		No change			↗ Increase													

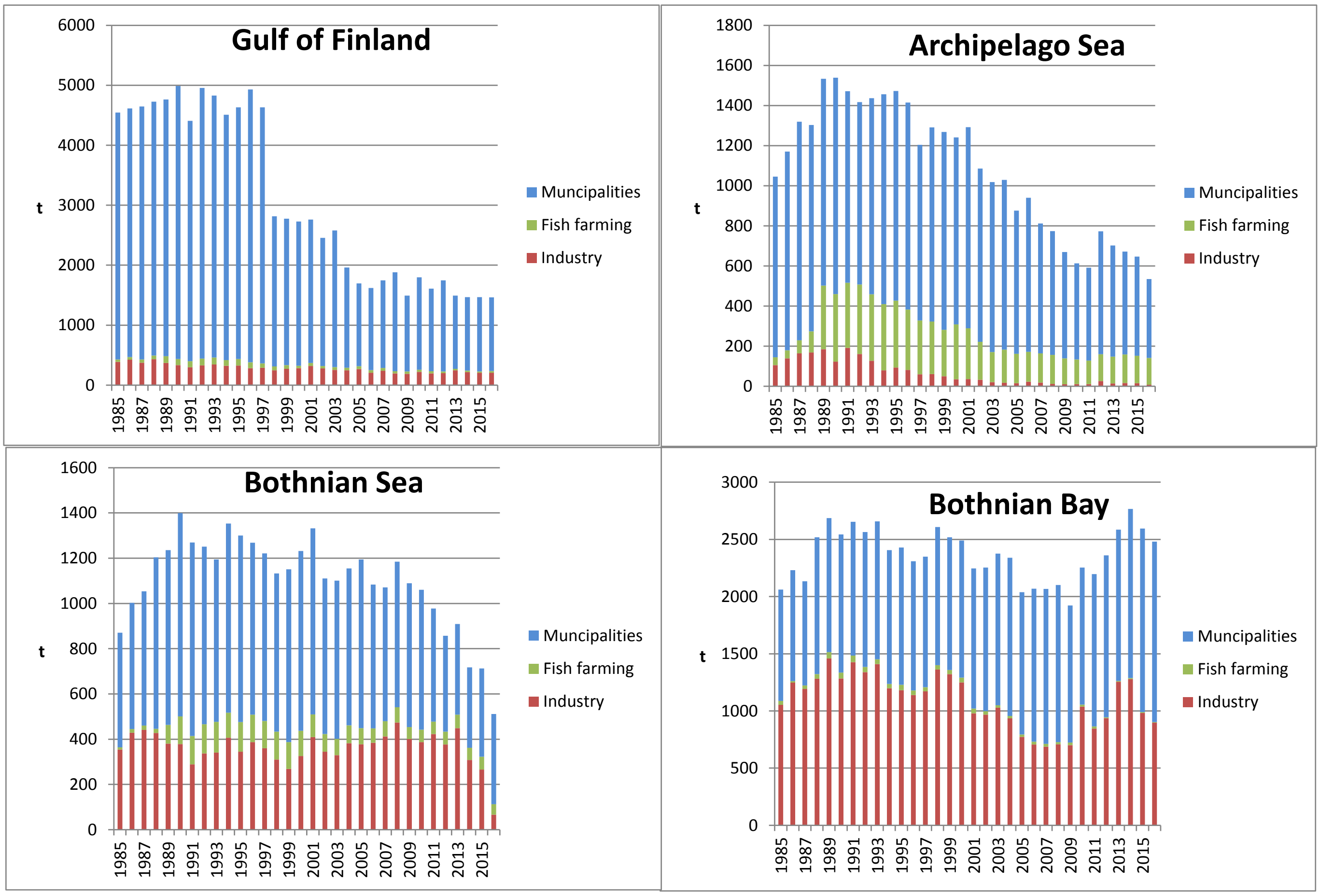

Fig. S1. Direct TN loads from point sources into different sea-regions from 1985 to 2016.

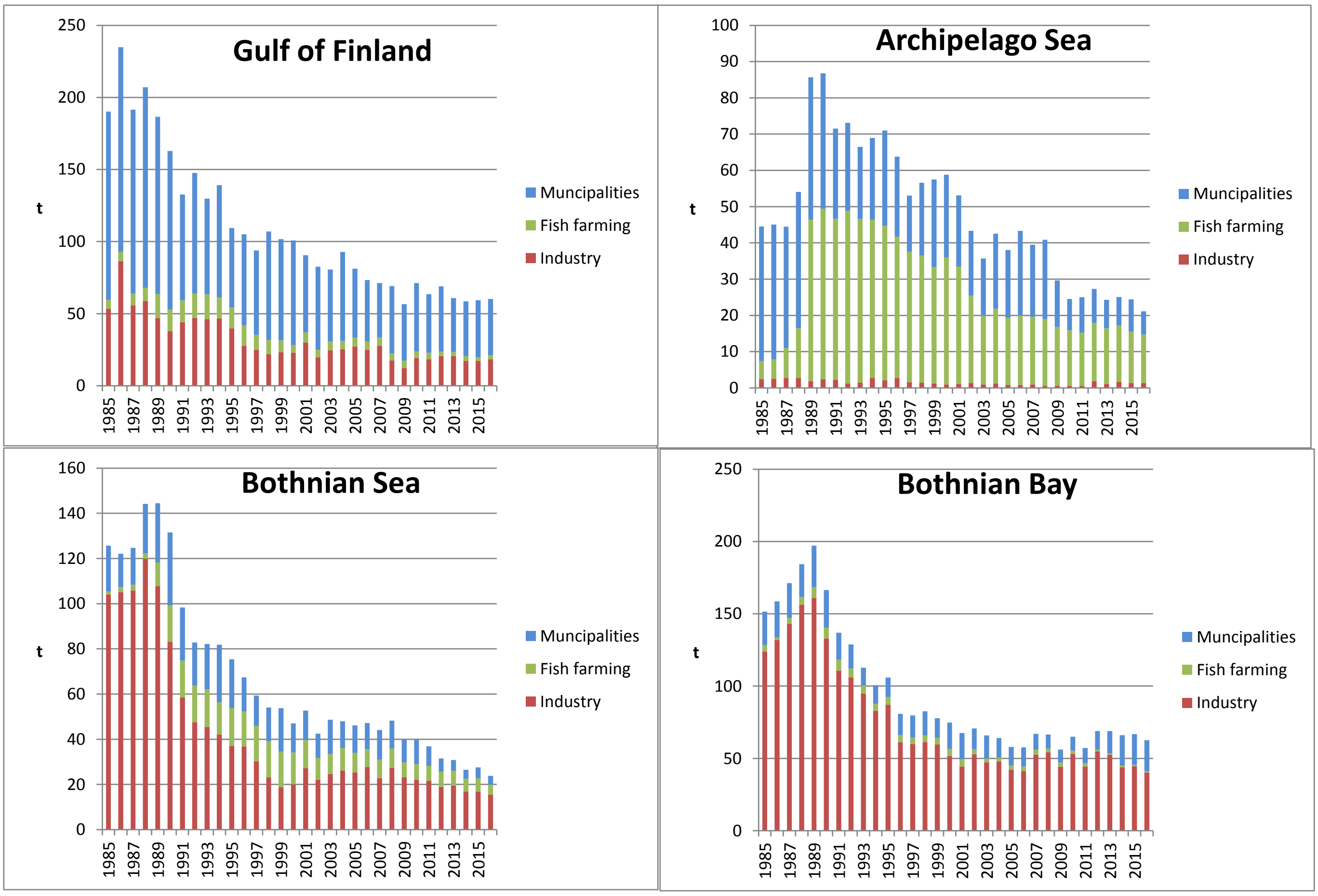

Fig. S2. Direct TP loads from point sources into different sea-regions from 1985 to 2016.

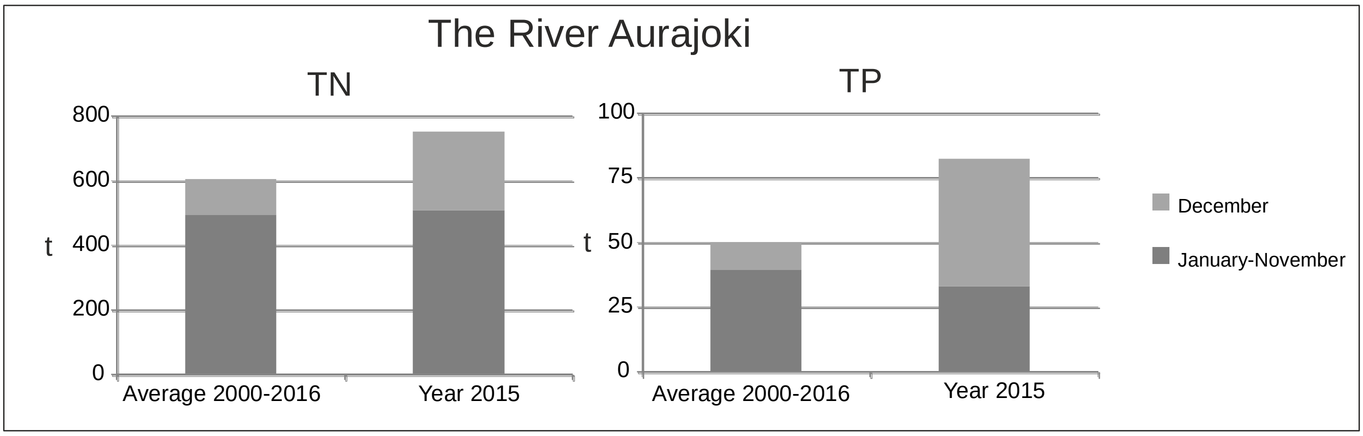

Figure S3. The average (years 2000−2016) TN and TP export in January-November and in December and the respective exports in 2015.
